# Malignancies Presenting With ANCA Positivity: Two Case Reports and Diagnostic Considerations

**DOI:** 10.1002/iid3.70238

**Published:** 2025-08-08

**Authors:** Min Zhao, Yue Lu, Ling Li

**Affiliations:** ^1^ Department of Rheumatology The First Affiliated Hospital of Dalian Medical University Dalian China

**Keywords:** ANCA‐associated vasculitides, case report, MPO ANCA, PR3 ANCA, tumor

## Abstract

**Background:**

ANCA‐associated vasculitides (AAV) are small‐vessel inflammatory disorders characterized by the presence of anti‐neutrophil cytoplasmic antibodies (ANCA), including those against proteinase 3 (PR3) and myeloperoxidase (MPO). ANCA positivity is not exclusive to vasculitis and may also occur in the context of malignancies or infections, leading to diagnostic confusion. In this report, we present two cases of malignancy with ANCA positivity.

**Case Presentation:**

The first case involved a 42‐year‐old male with nasopharyngeal symptoms, hearing loss, and proteinuria. Laboratory findings revealed elevated PR3‐ANCA and MPO‐ANCA levels, and a diagnosis of AAV was initially made. Despite immunosuppressive treatment, the patient developed persistent fever and rapid clinical deterioration. A subsequent bone marrow biopsy confirmed lymphoma with bone marrow infiltration. The second case presented with abdominal pain and retroperitoneal fibrosis. PET‐CT imaging identified multifocal soft tissue masses in the lacrimal glands, nasopharynx, retroperitoneum, thoracic‐lumbar vertebrae, and rib region. Histopathological analysis confirmed an inflammatory myofibroblastic tumor.

**Conclusion:**

These cases highlight the potential for tumors to mimic AAV both clinically and serologically. They emphasize the importance of a careful differential diagnosis in patients with ANCA positivity, particularly when clinical progression is atypical or treatment response is suboptimal. The pathophysiological relationship between tumors and AAV remains unclear, but may involve common mechanisms such as neutrophil extracellular trap formation and autoantigen presentation of PR3 and MPO. Further studies are needed to elucidate these links and improve diagnostic accuracy in such complex presentations.

## 1 Introduction

Anti‐neutrophil cytoplasmic antibodies (ANCA) are autoantibodies directed against cytoplasmic components of neutrophils and serve as important serological markers in the diagnosis of ANCA‐associated vasculitides (AAV) [1]. AAV encompasses a group of small‐vessel vasculitides, all of which may present with a broad spectrum of clinical manifestations [1]. These range from nonspecific systemic symptoms—such as fever, malaise, weight loss, and arthralgia—to organ‐specific involvement, including proteinuria, pulmonary infiltrates or nodules, sinusitis, interstitial lung disease, and cutaneous vasculitic lesions [2].

Despite its diagnostic utility, ANCA positivity is not pathognomonic for vasculitis and may also occur in non‐vasculitic conditions, including infections, drug reactions, and malignancies [3]. This overlap poses a significant diagnostic challenge in clinical practice, particularly when ANCA positivity is interpreted in isolation or in the setting of atypical clinical features. Among these, malignancies are of particular concern due to their potential to induce paraneoplastic autoimmune phenomena, including the production of ANCA through mechanisms that remain poorly understood. It has been hypothesized that tumor‐associated antigens may trigger aberrant immune responses, leading to ANCA generation. In this report, we describe two cases of ANCA positivity in patients ultimately diagnosed with malignant tumors.

### 1.1 Case 1 Presentation

A 42‐year‐old male presented to the otolaryngology (ENT) clinic 4 months earlier with complaints of nasal congestion for 6 months, dry throat, and progressive hearing loss for over a month. Nasal endoscopy revealed diffuse mucosal swelling, narrowing of the nasal cavity, and a smooth nasopharyngeal surface. Laryngoscopy showed scattered ulcerative lesions in the pharynx and thickening of the epiglottis. Sinus computed tomography (CT) indicated bilateral sinusitis with mucosal thickening of the inferior turbinates. Audiometric testing confirmed bilateral conductive hearing loss.

Routine blood tests were within normal limits, and liver and renal function were unremarkable. Urinalysis revealed proteinuria (2+) but no occult blood. Viral serology was negative. Due to the involvement of the nasal passages and ears, along with positive proteinuria, the patient was referred to a rheumatologist for further evaluation.

Serological testing showed elevated anti‐proteinase 3 antibodies (anti‐PR3, 33.47 U/mL; reference range: 0–20), anti‐myeloperoxidase antibodies (anti‐MPO, 49 U/mL; reference: 0–20), elevated erythrocyte sedimentation rate (ESR, 36 mm/h; normal: 0–15), and C‐reactive protein (CRP, 67.62 mg/L; normal: 0–8). Based on these findings, a diagnosis of ANCA‐associated vasculitis was made. The patient was started on oral methylprednisolone (28 mg once daily) and mycophenolate mofetil (0.5 g twice daily). Glucocorticoids were gradually tapered, and his symptoms initially improved.

On June 24, 2024, the patient was admitted with fever and sore throat as the primary complaints. Before admission, his medication regimen included methylprednisolone (8 mg daily) and mycophenolate mofetil (0.5 g twice daily). The patient continued these medications regularly until admission. Apart from the previously documented history, he had no other comorbidities, no family history of autoimmune diseases, and no smoking or alcohol use.

**Figure 1:**
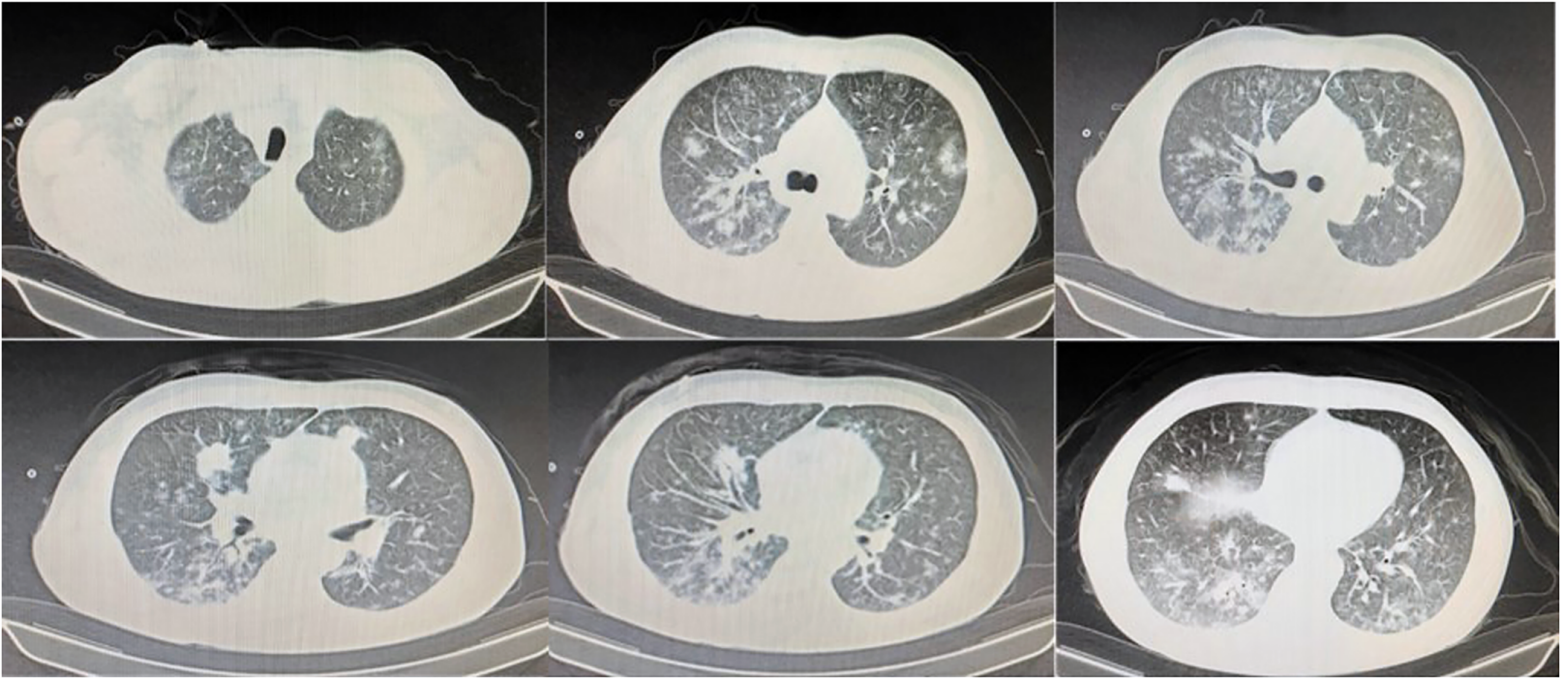
Chest CT imaging. Chest CT revealed scattered areas of inflammatory infiltration in both lungs.

Physical examination revealed an oral white pseudomembrane, conjunctival injection, and no palpable superficial lymphadenopathy. Laboratory testing showed negative ANCA titers, elevated serum Epstein‐Barr virus (EBV) DNA, and a sputum culture positive for *Staphylococcus aureus*. Chest CT demonstrated multiple nodular opacities of varying sizes in both lungs (Figure 1). Bronchoalveolar lavage with next‐generation sequencing (NGS) identified *S. aureus*, *Candida albicans*, and EBV. Flow cytometry of lymphocyte subsets revealed a decreased CD4+ T‐cell count (79 cells/μL) and an inverted CD4/CD8 ratio.

The patient was diagnosed with a pulmonary infection and initially treated with piperacillin‐tazobactam. Based on microbiological findings, antibiotics were escalated to vancomycin, and antifungal therapy with fluconazole was initiated. His fever subsided temporarily.

On hospital Day 9, the patient experienced a generalized seizure. Cranial CT and EEG were unremarkable. By hospital Day 11, he again developed fever, and laboratory tests showed thrombocytopenia (platelets: 63 × 10⁹/L). A multidisciplinary consultation suggested that thrombocytopenia could be due to infection or drug effects. The antibiotic regimen was adjusted to linezolid and fluconazole, and additional diagnostic workup, including bone marrow aspiration and PET‐CT, was recommended. However, the patient declined further evaluation.

Later that evening, he developed marked dyspnea and respiratory failure, necessitating transfer to the intensive care unit (ICU). Despite mechanical ventilation and broad‐spectrum antimicrobial coverage with vancomycin, caspofungin, meropenem, cotrimoxazole, and other agents, his condition continued to deteriorate. Progressive hematologic abnormalities developed, and serial laboratory results are summarized in Table 1.

**Table 1:** Main laboratory results and reference ranges during hospitalization of case 1.

Variable	Reference range	Day 1	Day 4	Day 8	Day 13	Day 19	Day 26
White blood cells (10^9/L)	3.50–9.50	4.33		3.57	3.47	1.36	0.1
Hemoglobin (g/L)	115–150	152		123	109	114	60
Platelet count (10^9/L)	125–350	89		123	58	40	13
Lymphocyte (10^9/L)	1.1–3.2	0.81		0.51	0.38	0.53	0.1
Lymphocyte (%)	20–50	18.7		14.3	11	39%	83.30%
Albumin (g/L)	40–55	23	20	30	22.3	27.5	29.3
Alanine aminotransferase (U/L)	7–40	43	58	110	80	251	89
aspartate aminotransferase (U/L)	13–35	101	78	72	133	360	94
Alkaline phosphatase (U/L)	50–135	266	135	129	334	443	244
Glutamyl transferase (U/L)	7–45	179	117	283	806	566	264
C‐reactive protein (mg/L)	≤ 10.00	85.66	—	—	—	—	—
Erythrocyte sedimentation rate (mm/h)	0–15	17	—	—	—	—	—
Fibrinogen (g/L)	1.8–3.9	1.83	1.06	1.1	1.52	2.29	3.39
d‐dimer (mg/L)	< 0.55	3.43	—	—	4.28	16.42	—
Procalcitonin (ng/mL)	≤ 0.5	0.25	—	—	—	—	—
EBV‐DNA	< 4E + 02	2.76E + 05	—	—	—	—	—
ANA	1: 100	0.111111111	—	—	—	—	—
pANCA	Negative	Negative	—	—	—	—	—
MPO‐ANCA (RU/mL)	＜20	Negative	—	—	—	—	—
cANCA	Negative	Negative	—	—	—	—	—
PR3‐ANCA	＜20	Negative	—	—	—	—	—
Urine protein quantification	0–150 mg/24 h	488 mg/24 h	—	—	—	—	—

Abbreviations: ALB, albumin; ALP, alkaline phosphatase; ALT, alanine aminotransferase; ANA, antinuclear antibody; AST, aspartate aminotransferase; cANCA, cytoplasmic anti‐neutrophil cytoplasmic antibodies; CRP, C‐reactive protein; d‐dimer, d‐dimer; EBV‐DNA, Epstein‐Barr virus DNA; ESR, erythrocyte sedimentation rate; FIB, fibrinogen; GGT, gamma‐glutamyl transferase; Hb, hemoglobin; Lym#, lymphocyte count; Lym%, lymphocyte percentage; MPO‐ANCA, myeloperoxidase anti‐neutrophil cytoplasmic antibodies; PCT, procalcitonin; PLT, platelet count; pANCA, perinuclear anti‐neutrophil cytoplasmic antibodies; PR3‐ANCA, proteinase 3 anti‐neutrophil cytoplasmic antibodies; Urine protein quantification, 24‐h urine protein measurement; WBC, white blood cells.

**Figure 2:**
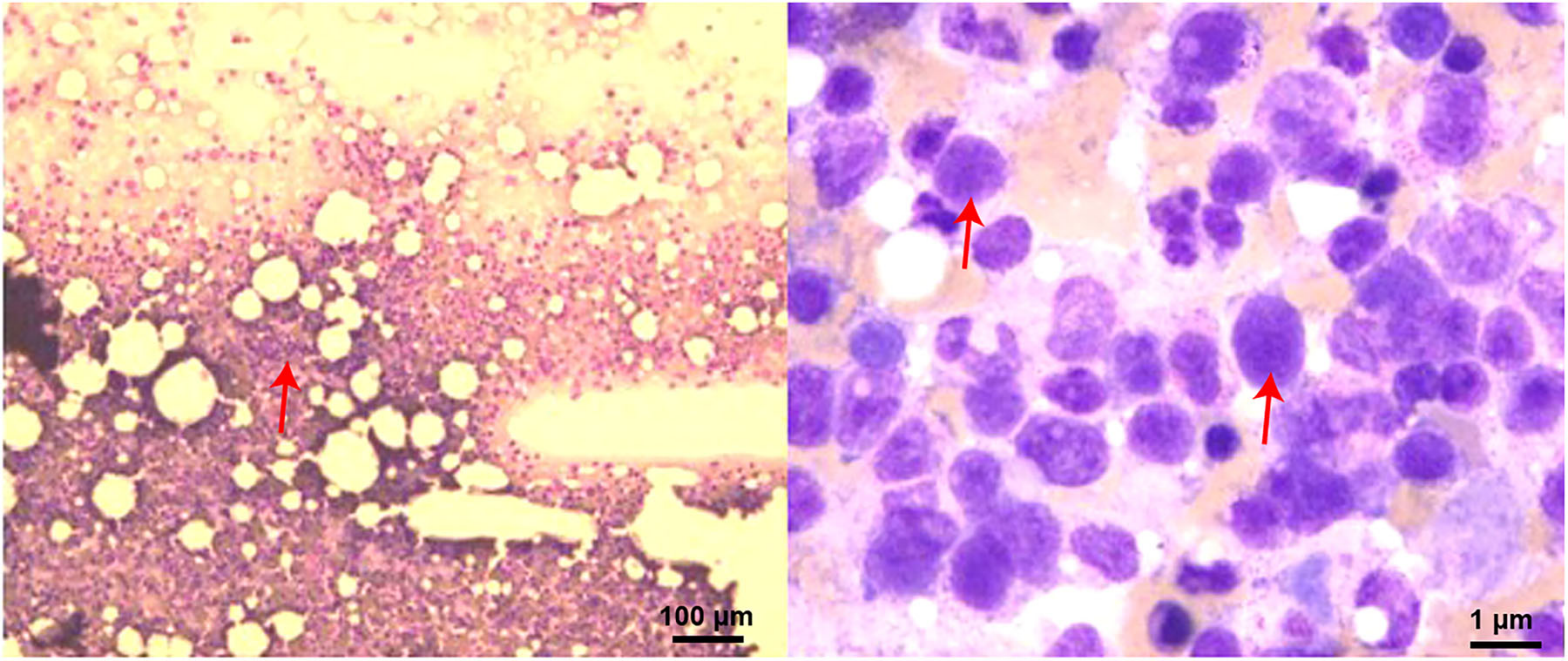
Bone marrow smear. The smear demonstrated lymphocytic infiltration of the bone marrow, with the presence of heterogeneous lymphocytes (left panel: red arrow indicates the area of bone marrow necrosis, magnification = 40×, scale bar = 100 µm; right panel: red arrow indicates the tendency of lymphoma cells, magnification = 100×, scale bar = 1 µm).

On Day 18 of hospitalization, complete blood counts showed pancytopenia. Bone marrow aspiration revealed lymphocytic predominance (54%), with 10% of the lymphocytes displaying abnormal morphology. These findings were suggestive of bone marrow infiltration by lymphoma (Figure 2). The diagnosis of lymphoma was subsequently confirmed.

Despite aggressive supportive care, the lymphoma complicated by severe systemic infection led to circulatory failure, and the patient passed away 3 days later. The timeline of this case was summarized in Supporting Information S1: Table S1.

### 1.2 Case 2 Presentation

A 76‐year‐old woman presented with persistent lower back and abdominal pain for more than 6 months, accompanied by fatigue and reduced appetite. She had unintentionally lost approximately 20 kg during this period. There were no associated symptoms such as fever, cough, sputum production, diarrhea, hematemesis, melena, urinary frequency, urgency, or dysuria. Both bowel movements and urination were normal.

A prior abdominal CTA performed at another hospital revealed a peri‐aortic mass. On August 15, 2024, the patient was admitted to our department for further evaluation. Initial laboratory tests (summarized in Table 2) showed: negative IgG4, positive pANCA, markedly elevated MPO‐ANCA (132.79 U/mL), elevated white blood cell count (WBC 16.35 × 10⁹/L), hemoglobin 98 g/L, platelets 214 × 10⁹/L, antinuclear antibody (ANA) 1:100, negative anti‐ENA and ds‐DNA, elevated CRP (189.52 mg/L) and ESR (74 mm/h), rheumatoid factor (RF) 23.6 IU/mL, IgG 22.71 g/L, IgM 4.12 g/L, IgA 1.98 g/L, and negative anti‐CCP.

**Table 2:** Summary of key laboratory results and reference ranges at admission (August 2024) for case 2.

Variables	Results
White blood cells (/L)	16.35 × 10^9^ (3.5–9.5)
Haemoglobin (g/L)	98 (115–150)
Platelet (/L)	214 × 10^9^ (125–350)
Albumin (g/L)	34.1 (40–55)
Creatinine (μmol/L)	58 (41–81)
ESR (mm/1 h)	74 (0–15)
CRP (mg/L)	189.52 (0–5)
Alanine aminotransferase (U/L)	15 (13–35)
Aspartate aminotransferase (U/L)	15 (7–40)
pANCA	Positive
MPO‐ANCA (RU/mL)	132.79 (< 20)
cANCA	Negative
PR3‐ANCA	Negative
ANA (fluorescence)	1:100 (1:＜100)
Immunoglobulin G (g/L)	22.71 (7–16)
Immunoglobulin A (g/L)	4.12 (0.7–4)
Immunoglobulin M (g/L)	1.98 (0.4–2.3)
Complement 3, complement 4	Normal
Carcinoembryonic antigen (ng/mL)	2.26 (0–5)
T‐spot	Negative

Abbreviations: ALB, albumin; ALT, alanine aminotransferase; ANA, antinuclear antibody; AST, aspartate aminotransferase; cANCA, cytoplasmic anti‐neutrophil cytoplasmic antibodies; CEA, carcinoembryonic antigen; CRP, C‐reactive protein; ESR, erythrocyte sedimentation rate; IgA, immunoglobulin A; IgG, immunoglobulin G; IgM, immunoglobulin M; MPO‐ANCA, myeloperoxidase anti‐neutrophil cytoplasmic antibodies; pANCA, perinuclear anti‐neutrophil cytoplasmic antibodies; PR3‐ANCA, proteinase 3 anti‐neutrophil cytoplasmic antibodies; T‐spot, T‐SPOT. TB test; WBC, white blood cells.

Contrast‐enhanced abdominal CT revealed extensive retroperitoneal fibrosis, and a diagnosis of idiopathic retroperitoneal fibrosis was initially considered. On the third day of hospitalization, a trial of methylprednisolone 40 mg daily for 3 days was administered to treat presumed inflammatory disease, but the patient showed no clinical improvement.

**Figure 3:**
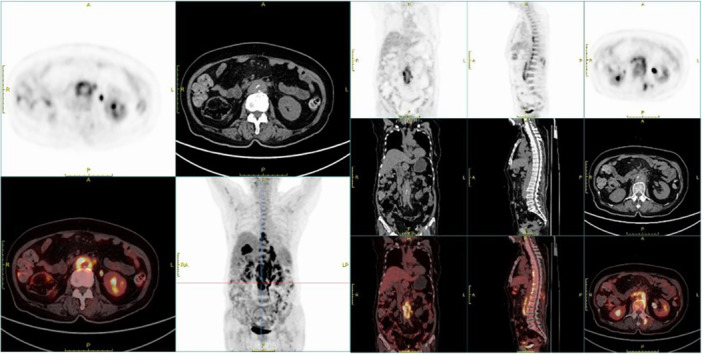
PET‐CT imaging. PET‐CT showed markedly increased fluorodeoxyglucose (FDG) uptake along the paraspinal region from the 7th thoracic vertebra to the third lumbar vertebra, as well as around the abdominal aorta at the level of the L1–L3 vertebrae.

Further evaluation with PET‐CT revealed multiple areas of increased fluorodeoxyglucose (FDG) metabolism, including the right lacrimal gland, musculature on the right side of the nasopharynx, slightly enlarged retroperitoneal lymph nodes, soft tissue density shadows near the right sixth rib, and along the T7–L3 vertebral levels, as well as around the abdominal aorta at the L1–L3 vertebral region (Figure 3). These findings raised suspicion for malignancy, and a pathological evaluation was recommended.

**Figure 4:**
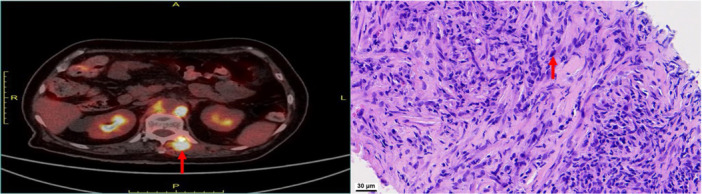
Histopathological findings. Left panel: Red arrow indicates the paravertebral tissue biopsy site. Right panel: Light microscopy revealed spindle cell neoplastic hyperplasia with inflammatory cell infiltration (red arrow indicates abnormal spindle cells; magnification = 40×, scale bar = 30 µm).

Subsequently, an X‐ray‐guided paraspinal tissue biopsy was performed. Histopathological examination revealed spindle cell neoplastic hyperplasia with inflammatory cell infiltration and interspersed invasion into the transverse muscle—findings consistent with an inflammatory myofibroblastic tumor (IMT) (Figure 4). Immunohistochemical staining showed: Vimentin (+++), AE1/AE3 (–), IgG (plasma cells +), IgG4 (scattered plasma cells +), S‐100 (–), Desmin (–), CD34 (–), Rb (+), MDM2 (–), CDK4 (–), CD138 (plasma cells +), P53 (5%), and Ki‐67 (10%). No histological features of vasculitis were observed, supporting the diagnosis of neoplasia rather than an inflammatory vasculitic process.

The elevated ANCA levels were considered to be paraneoplastic, likely related to the tumor. The patient was managed symptomatically with analgesics such as tramadol and oxycodone hydrochloride as needed. She opted to be discharged and passed away 4 months later. The timeline of this case was summarized in Supporting Information S1: Table S2.

## 2 Discussion

Two patients presented with elevated ANCA levels; however, neither met the full criteria for a diagnosis of ANCA‐associated vasculitis (AAV). ANCA positivity is not specific to AAV and can be observed in various other conditions, including infections, drug exposure, and even in healthy individuals [4, 5, 6]. In our study, the first patient was ultimately diagnosed with lymphoma, while the second was found to have an inflammatory myofibroblastic tumor. These cases highlight the importance of recognizing malignancy as a potential cause of ANCA positivity in clinical practice, especially given the unclear mechanisms linking tumors and ANCA.

Malignancies can present with vasculitis‐like symptoms, underscoring the importance of local pathological examination for accurate diagnosis. In our first case, earlier biopsy during the initial ENT evaluation could have potentially facilitated a timely diagnosis and altered the clinical course. Several reports have described tumor‐mimicking vasculitis, such as Kaposi's sarcoma, which presents with rash and positive pANCA and MPO‐ANCA, initially misdiagnosed as AAV. However, the condition worsened despite immunosuppressive therapy, and subsequent biopsy revealed HHV‐8‐positive Kaposi's sarcoma [7, 8, 9].

Although ANCA positivity and lymphoma can raise concern for paraneoplastic vasculitis, several features in Case 1 make primary AAV more plausible. His ENT‐limited symptoms and proteinuria began 4 months before any lymphoma evidence. Anti‐PR3 (33.5 U/mL) and anti‐MPO (49 U/mL) titers paralleled ESR/CRP levels and improved with glucocorticoid plus mycophenolate therapy, a pattern less typical of paraneoplastic AAV. By the time lymphoma was diagnosed, ANCA levels had fallen to undetectable while vasculitic signs remained quiescent. These findings suggest that vasculitis preceded malignancy rather than being directly driven by it.

ANCA positivity alone is insufficient for diagnosing AAV. In our case, chemiluminescence assays showed ANCA positivity, while immunofluorescence was negative, reinforcing the need for a comprehensive differential diagnosis. Even when AAV diagnostic criteria are met, the risk of underlying malignancy remains, especially when tumors initially mimic vasculitic symptoms.

One illustrative report involved a 62‐year‐old man with recurrent epistaxis, nasal congestion, and eyelid swelling. His initial pathology suggested IgG4‐related disease with a high IgG4 + /IgG ratio and elevated cANCA, PR3‐ANCA, and IgG4 levels, leading to a diagnosis of AAV with IgG4‐related features. Despite rituximab treatment, recurrent dacryocystitis prompted further investigation, and biopsy revealed HPV16‐positive squamous cell carcinoma of the lacrimal sac [10]. Similarly, Oba et al. reported a case of intravascular large B‐cell lymphoma presenting with vasculitis‐like symptoms and dual ANCA positivity, which was confirmed via renal biopsy. Treatment with R‐CHOP and intrathecal chemotherapy led to ANCA normalization and clinical improvement [11].

Our first patient shared many features with these cases, including vasculitis‐like symptoms and an initial transient response to glucocorticoids. Rapid deterioration occurred after 4 months, compounded by opportunistic infections and continued use of mycophenolate mofetil. Despite the absence of lymphadenopathy, nasopharyngeal involvement, and proteinuria warranted pathological investigation, thrombocytopenia required prompt bone marrow assessment. An additional limitation is the absence of a renal or nasal biopsy in Case 1, which limits histopathologic confirmation of vasculitis and may leave residual diagnostic uncertainty.

In Case 2, mass‐like lesions were detected in the paravertebral, retroperitoneal, spinal, lacrimal, and nasopharyngeal regions. Empiric glucocorticoid therapy was ineffective. Biopsy of the paravertebral lesion confirmed an inflammatory myofibroblastic tumor. Similar cases have been misdiagnosed as GPA [12]. These cases underscore the diagnostic challenges in differentiating tumor‐mimicking vasculitis, with histopathological confirmation being essential for accurate diagnosis.

There are reports documenting the coexistence of malignancy and AAV. In one case, a patient with chronic lymphocytic leukemia developed alveolar hemorrhage, and renal biopsy revealed atypical lymphocytes, crescentic glomerulonephritis, and pauci‐immune deposition [13]. Another case involved a breast cancer patient who developed acute kidney injury during preoperative evaluation. Renal biopsy revealed crescentic glomerulonephritis with pauci‐immune deposition, along with positive MPO‐ANCA and pANCA [14]. Although surgical resection and rituximab treatment were effective, tumor removal alone did not resolve the vasculitis symptoms.

A patient with gastric adenocarcinoma presented with MPO‐ANCA and pANCA positivity and vasculitis symptoms, including lower limb numbness and alveolar hemorrhage. MPO‐ANCA levels increased after tumor resection, but symptoms were later controlled with corticosteroids and rituximab [15]. Another case described a breast cancer patient with AAV who developed severe alveolar hemorrhage; following pulse glucocorticoid therapy and plasma exchange, tumor resection was performed without recurrence or metastasis [16]. In Case 2, the only abnormality suggestive of AAV was MPO‐ANCA positivity. The patient's periaortic mass and lack of any organ‐based vasculitic manifestations argue against true AAV. Instead, her presentation is best viewed as malignancy accompanied by MPO positivity. We propose that factors within the tumor microenvironment, such as heightened neutrophil extracellular trap formation and increased MPO antigen release, could drive ANCA production even in the absence of clinical vasculitis. Reports of malignancy‐related MPO‐ANCA without overt vasculitic features are scarce, underscoring the need for further research into these paraneoplastic antibody responses.

Tumorigenesis has been linked to AAV, although part of this association may result from cytotoxic treatments, particularly cyclophosphamide. Glucocorticoids and rituximab are now considered safer alternatives, as cumulative cyclophosphamide doses exceeding 36 g are a known risk factor for malignancy, while rituximab may reduce this risk [17, 18, 19].

Evidence suggests that ANCA production in tumor patients may contribute to malignancy development. For instance, in primary sclerosing cholangitis, anti‐PR3‐ANCA positivity is an independent risk factor for cholangiocarcinoma [20]. In another study of glomerular diseases with concurrent malignancy, 3.2% of renal biopsy patients had malignancy, and 12.2% were MPO‐ANCA positive, implicating antibody‐mediated mechanisms in tumor development [21].

Conversely, tumors may induce ANCA expression. A review of AAV and hematologic malignancies proposed that abnormal lymphocyte proliferation can generate autoimmune responses, resulting in paraneoplastic AAV [22]. In Case 1, we believe lymphoma contributed to PR3‐ and MPO‐ANCA positivity. The initial misdiagnosis of AAV and ineffective treatment highlight the complexity of such presentations.

ANCA targets antigens such as PR3 and MPO. PR3 is highly expressed in bone marrow precursors and leukemia cells and plays roles in cell proliferation, inflammatory regulation, and endothelial apoptosis. Overexpression due to defective PR3 degradation may contribute to both vasculitis and hematologic malignancy [22, 23].

Surface PR3 on tumor cells may act as an autoantigen, especially under immune surveillance failure, leading to ANCA production. PR3 is implicated in tumor progression and metastasis, and its overexpression may simultaneously promote ANCA generation and vasculitis pathogenesis. Clinical reports of ANCA positivity in Hodgkin's lymphoma and B‐cell lymphoma further support this mechanism [24, 25]. Tumor growth may enhance PR3 and MPO release, promoting ANCA production and triggering AAV. These findings suggest that AAV and cancer may share common pathogenic pathways, emphasizing the need for integrated diagnostic and therapeutic strategies.

## 3 Conclusion

Clinicians should maintain a high index of suspicion for neoplastic processes in patients labeled with AAV who exhibit unusual progression or resistance to standard immunosuppressive regimens. Highlighting potential connections between ANCA production and malignancy reinforces the importance of comprehensive diagnostic evaluation in all ANCA‐positive individuals.

## Author Contributions

**Min Zhao:** writing – review and editing, writing – original draft, conceptualization, methodology, and software. **Yue Lu:** conceptualization, methodology, software, writing – review and editing, and writing – original draft. **Ling Li:** investigation, conceptualization, methodology, validation, formal analysis, supervision, data curation, writing – original draft, writing – review and editing.

## Ethics Statement

This study was reviewed and approved by the Ethics Committee of the First Affiliated Hospital of Dalian Medical University.

## Consent

All patients provided written informed consent to participate in the study and for their data to be published.

## Conflicts of Interest

The authors declare no conflicts of interest.

## Supporting information


**Supporting Table S1:** Timeline of Case 1. **Supporting Table S2:** Timeline of Case 2.

## Data Availability

Data will be made available upon reasonable request.
