# Supplementary material for: Malignancies Presenting With ANCA Positivity: Two Case Reports and Diagnostic Considerations
Source: Immun Inflamm Dis. 2025 Aug 8;13(8):e70238. doi: 10.1002/iid3.70238 (PMC12332532; doi:10.1002/iid3.70238)
Supplement: Supplementary file 1 — Supporting Table S1: Timeline of Case 1. Supporting Table S2: Timeline of Case 2. [file IID3-13-e70238-s001.docx]

**Supplememtal Table S1. Timeline of Case 1**

| **Date** | **Interval/Day** | **Event** |
| --- | --- | --- |
| ~Dec 2023 | 6 months before | Onset of nasal congestion and dry throat |
| Feb 2024 | 4 months before | Progressive hearing loss → ENT evaluation |
| Early 2024 | — | Rheumatology workup: PR3-ANCA 33.5 U/mL, MPO-ANCA 49 U/mL, ESR 36 mm/h, CRP 67.6 mg/L; diagnosed AAV; started methylprednisolone + MMF |
| June 24, 2024 | Admission Day 1 | Fever, sore throat; oral pseudomembrane, conjunctivitis; chest CT w/ lung nodules; treated as pulmonary infection |
| July 3, 2024 | Day 9 | Generalized seizure |
| July 5, 2024 | Day 11 | Recurrent fever; thrombocytopenia (PLT 63 ×10⁹/L); antibiotic regimen adjusted |
| July 11, 2024 | Day 18 | Bone marrow aspiration → lymphocytic predominance suggesting lymphoma |
| July 14, 2024 | Day 21 | Death from circulatory failure secondary to lymphoma with severe infection |

**Supplememtal Table S2. Timeline of Case 2**

| **Date** | **Interval/Day** | **Event** |
| --- | --- | --- |
| Feb–Mar 2024 | > 6 months before | Onset of lower back & abdominal pain, fatigue, 20 kg weight loss |
| Aug 15, 2024 | Admission Day 1 | Admitted: MPO-ANCA 132.8 U/mL, CRP 189.5 mg/L, ESR 74 mm/h; CT → retroperitoneal fibrosis; trial of methylprednisolone 40 mg×3 days—no improvement |
| Aug 18, 2024 | Day 3 | PET-CT → multifocal FDG-avid masses (lacrimal glands, nasopharynx, retroperitoneum, spine, ribs) |
| Aug 20, 2024 | ~Day 5 | CT-guided paraspinal biopsy → spindle cell neoplastic hyperplasia + inflammatory infiltrate → diagnosis: IMT |
| Late Aug 2024 | — | Symptomatic management with tramadol/oxycodone; patient opts for discharge |
| Dec 2024 | ~ 4 months later | Patient passes away |
